# Simple propagation method for resident macrophages by co-culture and subculture, and their isolation from various organs

**DOI:** 10.1186/s12865-019-0314-z

**Published:** 2019-09-18

**Authors:** Kazushige Ogawa, Mayu Tsurutani, Aya Hashimoto, Miharu Soeda

**Affiliations:** Laboratory of Veterinary Anatomy, Graduate School of Life and Environmental Sciences, Osaka Prefecture University, 1-58 Rinku-Ourai-Kita, Izumisano, Osaka, 598-8531 Japan

**Keywords:** Resident macrophages, Cell culture, Kupffer cells, Red pulp macrophages, Lung interstitial macrophages, Microglia

## Abstract

**Background:**

Resident macrophages (Mø) originating from yolk sac Mø and/or foetal monocytes colonise tissues/organs during embryonic development. They persist into adulthood by self-renewal at a steady state, independent of adult monocyte inputs, except for those in the intestines and dermis. Thus, many resident Mø can be propagated in vitro under optimal conditions; however, there are no specific in vitro culture methods available for the propagation of resident Mø from diverse tissues/organs.

**Results:**

We provided a simple method for propagating resident Mø derived from the liver, spleen, lung, and brain of ICR male mice by co-culture and subculture along with the propagation of other stromal cells of the respective organs in standard culture media and successfully demonstrated the propagation of resident Mø colonising these organs. We also proposed a simple method for segregating Mø from stromal cells according to their adhesive property on bacteriological Petri dishes, which enabled the collection of more than 97.6% of the resident Mø from each organ. Expression analyses of conventional Mø markers by flow cytometry showed similar expression patterns among the Mø collected from the organs.

**Conclusion:**

This is the first study to clearly provide a practical Mø propagation method applicable to resident Mø of diverse tissues and organs. Thus, this novel practical Mø propagation method can offer broad applications for the use of resident Mø of diverse tissues and organs.

## Background

Macrophages (Mø) are heterogeneous and multifunctional cells that are indispensable for the development and regeneration of tissues and organs, and also assist in the removal of pathogens invading the body. In adults, Mø are largely divided into two types: (1) resident Mø, which colonise tissues/organs at a steady state and perform tissue/organ-specific functions to maintain tissue homeostasis, and (2) recruited Mø or bone-marrow derived Mø, which differentiate from circulating monocytes in the blood infiltrating lesions in response to damage of tissues/organs. Resident Mø in adults were previously considered to originate from the bone marrow-derived monocytes that are gradually replaced by monocyte-derived Mø, which undergo tissue/organ-specific differentiation. Thus, resident Mø were regarded as the terminally differentiated cells that did not proliferate locally in colonising tissues in a steady state. However, evidence accumulated in the past decade has provided new insight into the origins of resident Mø, revealing that Mø in the yolk sac and/or foetal monocytes in the liver migrate to diverse tissues/organs during embryonic development, colonise, and undergo tissue/organ-specific differentiation into resident Mø locally. Moreover, most resident Mø persist into adulthood by self-maintenance of local proliferation processes in a steady state, independent of any input from bone marrow-derived monocytes, except for those colonising the intestines and dermis [1–5]. Furthermore, the cytokines that promote the self-renewing proliferation of resident Mø in the steady state have been identified, including colony stimulating factor 1 (CSF-1) for Kupffer cells, red pulp Mø, and other resident Mø; colony stimulating factor 2 (CSF-2) for alveolar Mø; and interleukin (IL)-34 for microglia [1, 5–7]. These findings suggest that most resident Mø have the capacity to proliferate in vitro under suitable conditions, which opens up the possibility of obtaining a large number of resident Mø for a variety of research applications, including those currently utilising adult monocyte-derived Mø.

In vitro methods are now well established for the collection of monocytes from the blood, bone marrow, and spleen; their temporary propagation and differentiation into Mø; and the activation/polarisation of adult monocyte-derived Mø. Standardised experimental guidelines for activation of classical (M1) and alternative (M2) monocyte-derived Mø states have also been developed [8], as these polarisations are key responses and factors that determine lesion development, progression, and regression in diverse diseases [9, 10]. Moreover, isolation methods have been established for resident Mø from several organs such as the brain (microglia), liver (Kupffer cells), and lung (alveolar Mø). It is also now possible to culture and temporarily proliferate a few of these resident Mø from the respective organs, including microglia [11–13] and Kupffer cells [14]. Specifically, proliferated microglia and Kupffer cells appear as low-adhesive round cells in primary co-culture with organ-specific stromal cells and can thus be collected as floating cells by relatively simple physical separation methods such as shaking or tapping. However, the total number of cells that can be obtained with these methods is limited owing to their inadequate proliferation and separation in primary culture. Thus, ex vivo methods are still primarily used for studies of both resident and recruited Mø, such as those aiming to examine their behaviours in lesions, including Mø polarisations. However, it is difficult to clearly distinguish between resident and recruited Mø in lesions because they share common molecular markers [15, 16]. Hence, an improved in vitro method is needed to advance research on the specific behaviours of resident Mø in diverse tissues/organs in response to various cytokines and molecules produced by pathogens such as lipopolysaccharide (LPS).

Guilliams and Scott [7] recently proposed that resident Mø nourished under suitable niches have self-renewal properties and can undergo organ-specific differentiation. Therefore, we hypothesised that resident Mø in a certain organ could be propagated along alongside the propagation of niche-forming cells residing in the respective organ. This could overcome the current limitation of the subculture of resident Mø that tend to adhere to the culture treatment, requiring harsh treatment conditions. Toward this end, we developed a simple propagation method that can be commonly applied to resident Mø. In brief, the method involves propagation of Mø in co-culture with other stromal cells of specific organs/tissues, followed by subculture and isolation on the basis of their adhesive property to bacteriological Petri dishes using a standard culture medium. To the best of our knowledge, this is the first report of a method for propagating several resident Mø by subculture. Using the proposed method, we were able to propagate specific Mø derived from the adult mouse liver, spleen, and lung, as well as those derived from the pubertal mouse brain in quantities sufficient to be used in a variety of research applications.

## Results

### Propagation behaviour of co-cultured resident macrophages

**Fig. 1:**
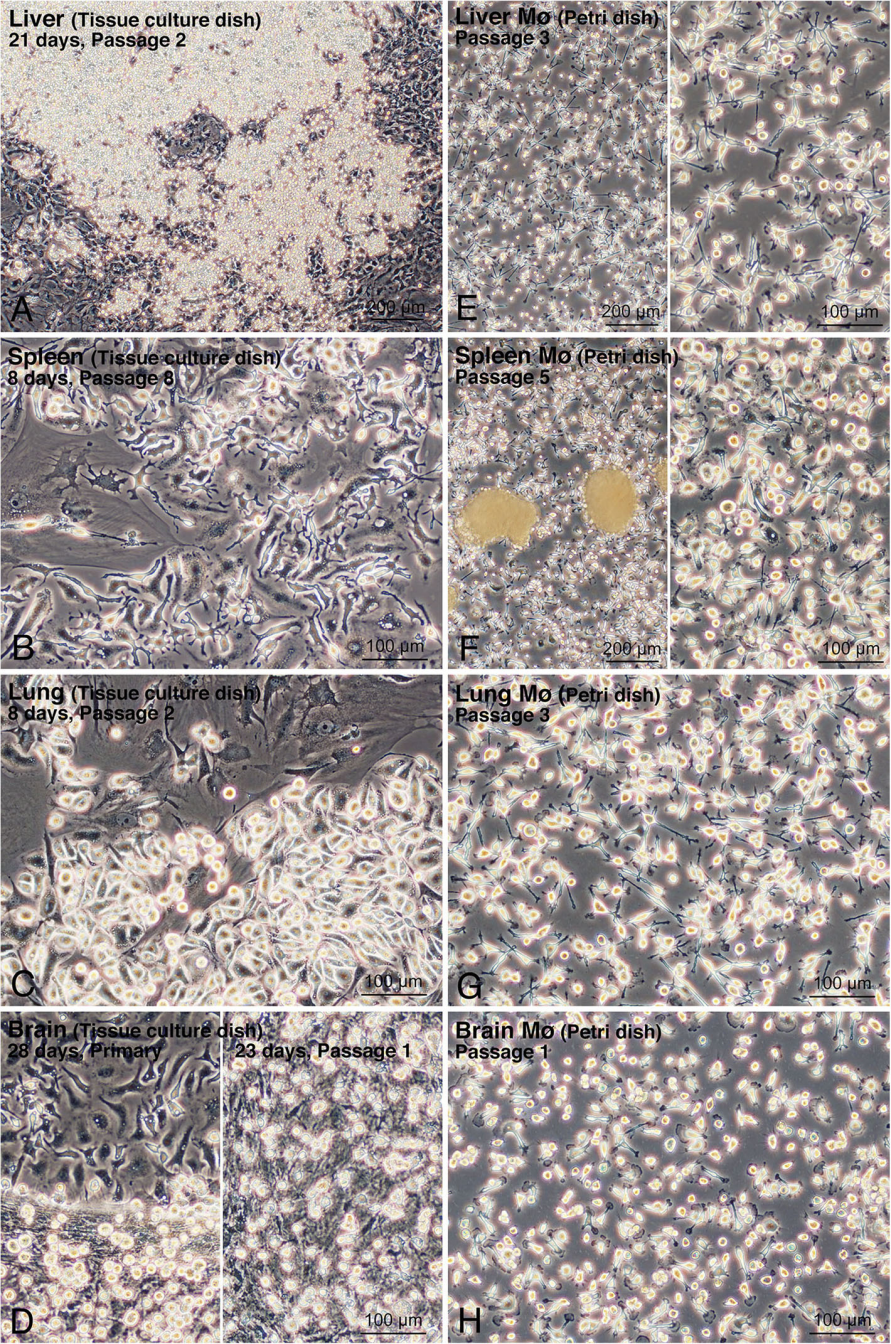
Propagation of liver, spleen, lung, and brain Mø in co-culture and subculture with their respective stromal cells in tissue culture dishes and their segregation using bacteriological Petri dishes. **a**: Liver cells after passage 2, 21 days after seeding in a tissue culture dish. **b**: Spleen cells after passage 8, 8 days after seeding in a tissue culture dish. **c**: Lung cells after passage 2, 8 days after seeding in a tissue culture dish. **d**: Primary brain cells 28 days after seeding (left panel) and brain cells after passage 1, 23 days after seeding (right panel) in a tissue culture dish. **e–h**: Liver, spleen, lung, and brain Mø in bacteriological Petri dishes. Mø selectively adhered to the dish surface and non-adherent cells formed cell aggregates. Cell aggregates were removed by washing with conditioned medium except for the left panel of F. **e**: Liver Mø segregated from liver stromal cells after passage 3 of subculture. **f**: Spleen Mø segregated from spleen stromal cells after passage 5 of subculture. **g**: Lung Mø segregated from lung stromal cells after passage 3 of subculture. **h**: Brain Mø segregated from brain stromal cells after passage 1 of subculture

Mø derived from the mouse liver, spleen, lung, and brain showed high propagation when co-cultured with stromal cells of the respective organs in standard culture media [Dulbecco’s modified Eagle’s medium (DMEM) for the liver, spleen, and lung, and DMEM/F12 for the brain] including 10% foetal bovine serum (FBS) without any additional growth factors for Mø such as CSF-1 and CSF-2. By changing the culture media every 4–6 days, primary stromal cells, including Mø, reached over-confluence usually within 2–3 weeks for liver, spleen, and lung cells, and within 3–4 weeks for brain cells. The over-confluent cells formed a multi-layered structure on a standard tissue culture dish. The cells were then subcultured until reaching over-confluence again, which occurred within a similar period of time. The cells were considered to be Mø (designated as cMø) according to observations of their propagation behaviours by microscopy, which were similar among the cells cultured from the four organs (Fig. 1a–d). Within ten days after seeding, there were three morphological types of cMø apparent on the dish: flat cells with a few long processes adhering to large stromal cells, elongated cells with a few long processes adhering directly to the dish surface in a relatively low cell density area, and round or fusiform cells in a densely populated area on the dish surface (Fig. 1b, c). Two to three weeks after the subculture, when stromal cells became over-confluent, there were two morphological types of cMø observed in multi-layered cells: round, small cells located in the top layer, adhering to stromal cells forming the middle cell layer; and fusiform cells with a few long processes adhering directly to the dish surface, in which the stromal cells forming the middle cell layer unexpectedly detached from the dish surface (Fig. 1a, d).

Mø and stromal cells in co-culture derived from the liver, spleen, and lung that were subcultured for more than eight passages propagated and became over-confluent as observed for the primary cells, whereas cells from the brain showed a remarkable decrease in propagation after more than three passages. The over-confluent co-cultured cells were subcultured or frozen at a cell dilution ratio of 1:3 for the liver, spleen, and lung and at 1:2 for the brain. The thawed and cultured frozen cells were treated to the same cultivating condition. Frozen cells from the liver, spleen, and lung propagated similar to the unfrozen cells, whereas those from the brain propagated slowly and were therefore not suitable for Mø propagation.

### Macrophage segregation by adhesion to the bacteriological Petri dish

The Mø were separated from the other stromal cells in co-culture according to their adhesive property to bacteriological Petri dishes, in which only the Mø should adhere to the dish. Within a few days of seeding co-cultured over-confluent cells, small round cells with a few processes, i.e. Mø adhering to the dish surface, were observed; cells of other shapes rarely adhered to the dish, and cell aggregates floating in the media were also evident (Fig. 1e–h). These cell aggregates were easily removed by washing with conditioned media. The cell density of Mø was almost unchanged, with or without cell aggregates, in the dishes when culture continued for several days. We usually collected more than 1.5 × 10^6^ adherent cells per 10-cmø bacteriological Petri dish.

**Fig. 2:**
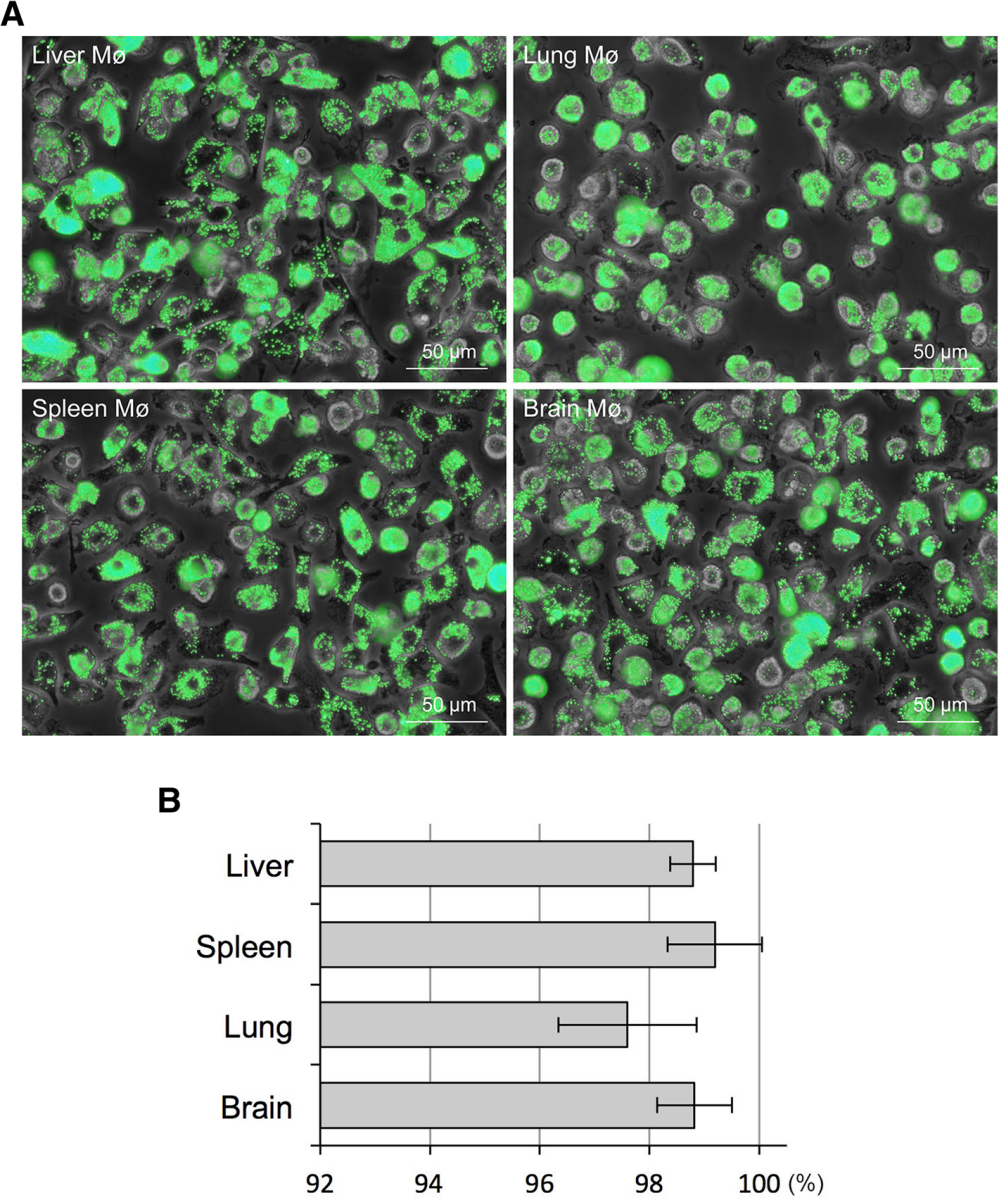
Mø content (%) in cells segregated by adhesion to bacteriological Petri dishes assessed by the phagocytosis of fluorescent beads. Cells adherent to the Petri dish were incubated with fluorescent beads, with an average diameter of 1.0 μm, for 2 h and then fixed. Phase-contrast images and green fluorescence images of the same fields were captured. **a**: Representative fluorescence images merged with phase-contrast images showing fluorescent beads phagocytosed by liver, spleen, lung, and brain Mø propagated by co-culture and subculture. **b**: More than 700 cells per sample were calculated, and the percentage of Mø in each organ (liver, spleen, lung, brain) derived from more than four mice was determined from more than four independent experiments. Bar graphs showing the percent content of Mø as the mean ± SD (liver, 98.8 ± 0.4; spleen, 99.2 ± 0.9; Lung, 97.6 ± 1.3; brain 98.8 ± 0.7)

Phagocytosis of fluorescent beads was evaluated to precisely determine the percentage of Mø in the collected segregated cells. During incubation, almost all of the cells segregated from the liver, spleen, lung, and brain stromal cells phagocytosed the fluorescent beads (Fig. 2a). Most of the cells had numerous beads in their cytoplasm, and the cytoplasm of some cells was completely filled with beads. This demonstrated that the Mø propagated in co-culture possess a high phagocytic property. The bead-positive and negative cells were counted to estimate the percentage of Mø in the segregated cells. We defined cells phagocytosing more than two beads as bead-positive cells and counted more than 700 cells per sample. Overall, these cells comprised more than 98.8% Mø from the liver, spleen, and brain as well as more than 97.6% Mø from the lung (Fig. 2b). Thus, Mø segregation according to their property of adhesion to the bacteriological Petri dish represents a simple method to purify Mø from stromal cells from various organs in co-culture.

### Expression profiles of macrophage markers by flow cytometry

**Fig. 3:**
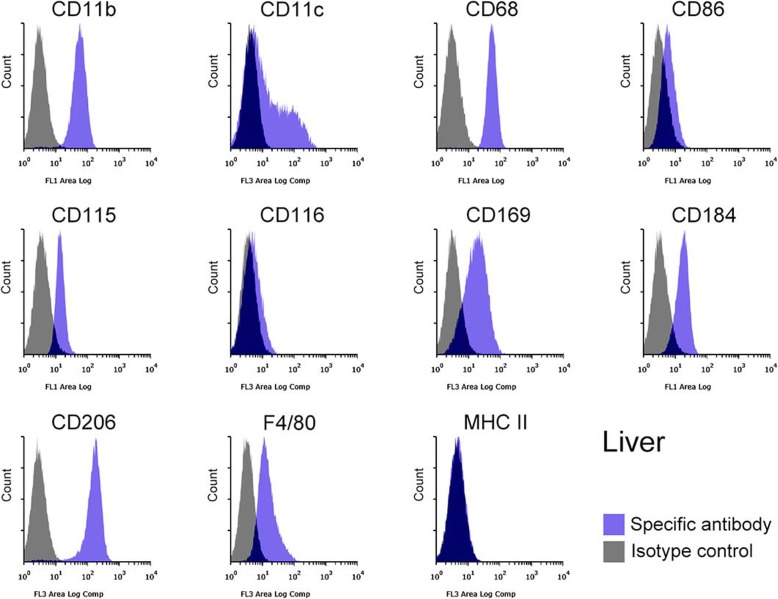
Representative histograms from flow cytometric analyses, showing the expression of CD11b, CD11c, CD68, CD86, CD115, CD116, CD169, CD184, CD206, F4/80, and MHC II as Mø markers in the liver Mø propagated in co-culture and by subculture (blue histogram, specific antibody; grey histogram, isotype control). Cell suspensions were pre-treated with the anti-mouse CD16/32 antibody and then treated with the fluorochrome-labelled test antibody or the same amount of fluorochrome-labelled isotype control antibody. All molecules are clearly expressed except for CD116 and MHC II. Histograms show a single peak except for those of CD11c, in which CD11c-positive and -negative fractions appear

**Fig. 4:**
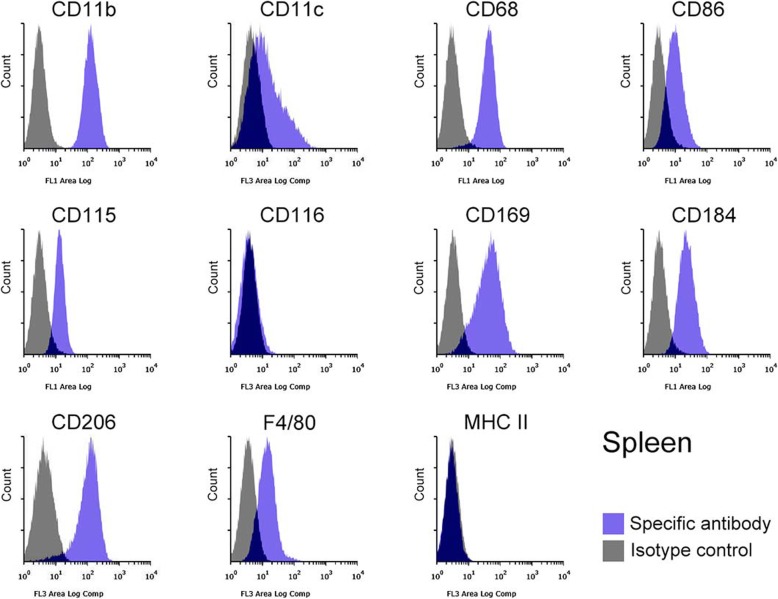
Representative histograms from flow cytometric analyses, showing the expression of CD11b, CD11c, CD68, CD86, CD115, CD116, CD169, CD184, CD206, F4/80, and MHC II as Mø markers in the spleen Mø propagated in co-culture and by subculture (blue histogram, specific antibody; grey histogram, isotype control). All molecules are clearly expressed except for CD116 and MHC II. Histograms show a single peak except for those of CD11c, in which CD11c-positive and -negative/-faintly positive/ fractions appear

**Fig. 5:**
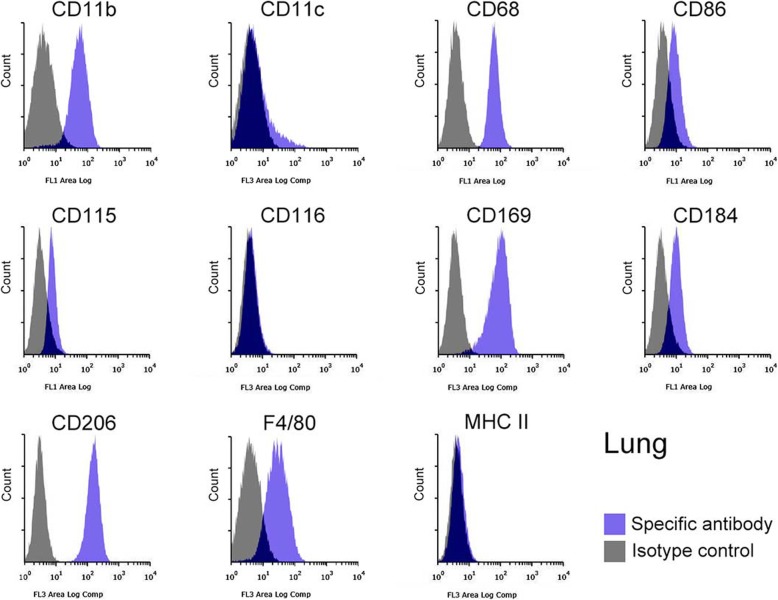
Representative histograms from flow cytometric analyses, showing the expression of CD11b, CD11c, CD68, CD86, CD115, CD116, CD169, CD184, CD206, F4/80, and MHC II as Mø markers in the lung Mø propagated in co-culture and by subculture (blue histogram, specific antibody; grey histogram, isotype control). All molecules are clearly expressed except for CD116, MHC II, and CD11c, whose histogram includes a small positive and a large negative fraction

**Fig. 6:**
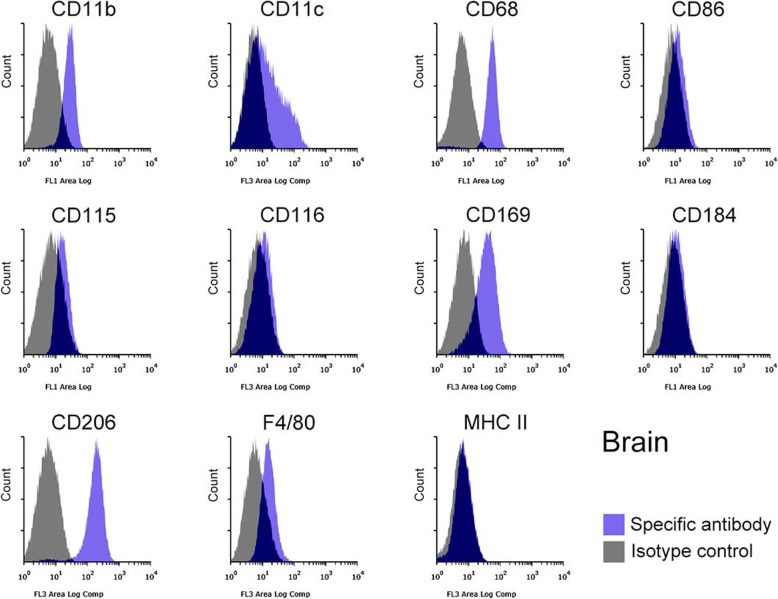
Representative histograms from flow cytometric analyses, showing the expression of CD11b, CD11c, CD68, CD86, CD115, CD116, CD169, CD184, CD206, F4/80, and MHC II as Mø markers in the brain Mø propagated in co-culture and by subculture (blue histogram, specific antibody; grey histogram, isotype control). CD11b, CD11c, CD68, CD169, CD206, and F4/80s are clearly expressed, while CD86, CD115, and CD116 are faintly expressed. Histograms show a single peak except for those of CD11c, in which CD11c-positive and -negative fractions appear

The identity of the Mø segregated from subcultured liver, spleen, lung, and brain stromal cells was further confirmed based on the expression of Mø markers using flow cytometry: integrin αM subunit (CD11b), integrin αX subunit (CD11c), scavenger receptor class D (CD68), CD86 (B7–2), CSF-1R (CD115), CSF-2R (CD116), Siglec-1 (CD169), C-X-C chemokine receptor type 4 (CD184), and C-type mannose receptor 1 (CD206), EGF-like module-containing mucin-like hormone receptor-like 1 (F4/80), and major histocompatibility complex class II (MHC II). The liver, spleen, lung, and brain Mø consisted of a single population based on histograms of the marker expression distribution (Figs. 3, 4, 5, 6): all histograms showed a single peak except for those of CD11c, which included both CD11c-positive and -negative fractions. Moreover, the respective Mø showed similar expression patterns of these molecules, and the patterns were quite similar between the liver and spleen Mø. As a whole, all Mø showed high expression of CD11b, CD68, CD169, and CD206; substantial or high expression of CD86, CD115, CD184, and F4/80, except for faint expression of CD86, CD115, and CD184 in the brain Mø; and faint or almost negative expression of CD116 and MHC II. These expression analyses clearly confirmed that the cells segregated from the co-culture of all four organs were Mø.

### M1/M2 polarisation induction of macrophages

We further examined whether polarisation of the segregated Mø to classical M1 and alternative M2 Mø occurred in response to stimulation with cytokines and the Toll-like receptor ligand. We used the spleen Mø as a representative of the four Mø. The combination of LPS and interferon-γ (IFN-γ) was used as an inducer for M1 polarisation, and IL-4 was used as an inducer for M2 polarisation as previously reported for monocyte-derived Mø [8, 17]. We also employed CD11c and CD86 as M1 polarisation markers, and CD206 as an M2 polarisation marker as well as CD11b as a control pan-Mø marker according to previous reports [18, 19].

**Fig. 7:**
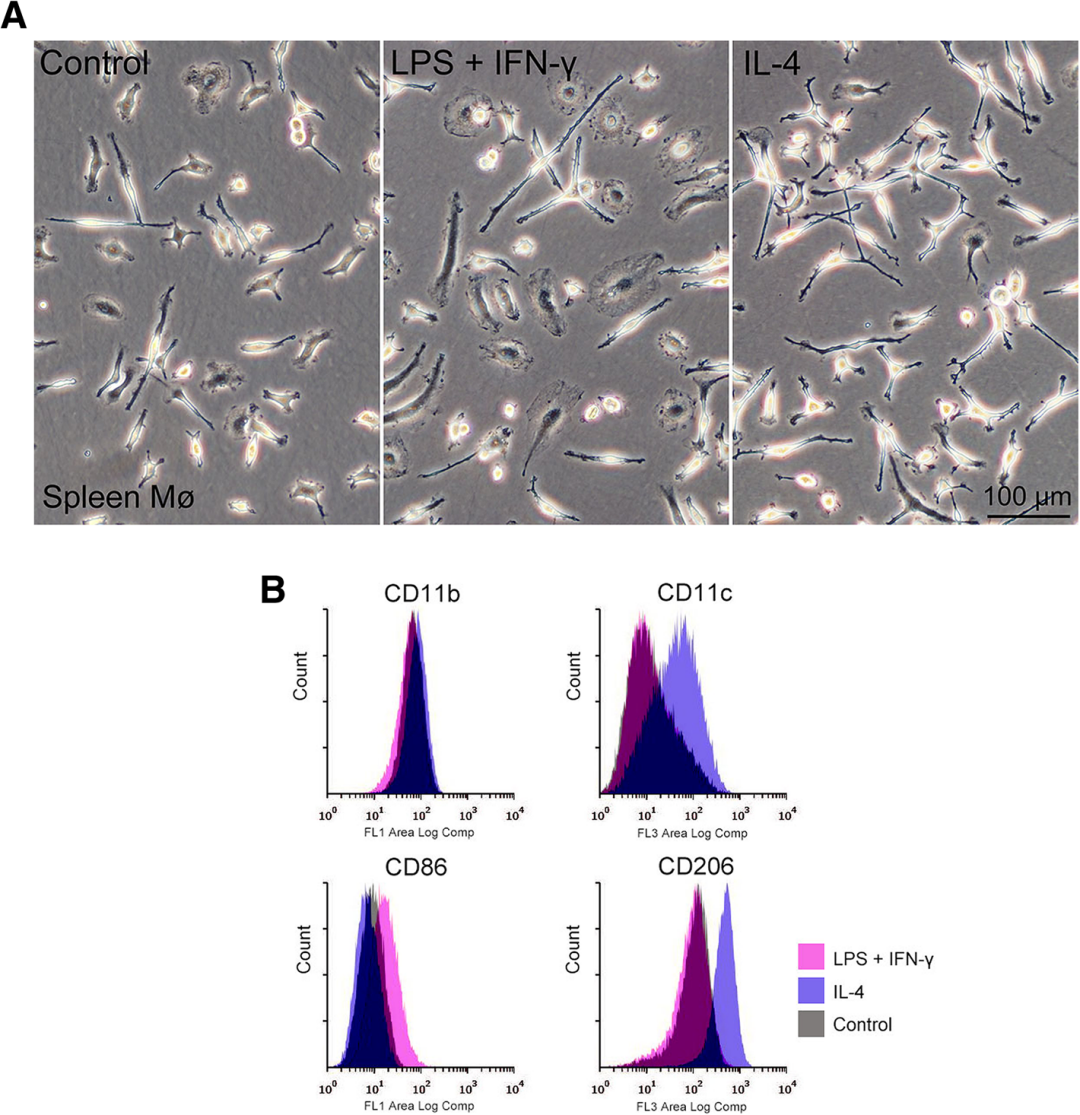
Morphology and expression levels of M1 and M2 polarisation markers in spleen Mø treated with M1 and M2 polarisation inducers. Spleen macrophages were treated with or without (control) LPS plus IFN-γ (M1 inducer) and IL-4 (M2 inducer) for 24 h. **a**: Representative phase-contrast images of spleen Mø treated with or without the inducers. **b**: Representative histograms from flow cytometric analyses, showing the expression of CD11b (pan Mø marker), CD11c and CD68 (M1 polarisation markers for monocyte-derived Mø), and CD206 (M2 polarisation marker for monocyte-derived Mø)

Flattened and/or elongated Mø frequently appeared on the bacteriological Petri dish after treatment with LPS plus IFN-γ for 24 h, while thin elongated Mø (with few long processes) occupied the dish after treatment with IL-4 (Fig. 7a). Flow cytometry revealed that LPS plus IFN-γ clearly upregulated CD86 expression, while IL-4 clearly upregulated CD11c and CD206 expression (Fig. 7b). These findings indicated that the propagated spleen Mø responded to the typical M1 and M2 polarisation inducers. Moreover, the polarisation properties likely differ between spleen and monocyte-derived Mø because CD11c is used as an M1 polarisation marker [18, 19]. Further, the cell adhesion capacity of the spleen Mø most likely increased in response to the M2 inducers because CD11c is an integrin αX subunit, which together with the ß2 subunit binds to integrin ligands such as ICAM-1, fibrinogen, and collagen [20].

## Discussion

We successfully propagated Mø collected from the mouse liver, spleen, lung, and brain after co-culture with stromal cells of the respective organs followed by subculture in standard culture media without any additional growth factors. These Mø were isolated from stromal cells according to their adhesive property to bacteriological Petri dishes. These propagated Mø were confirmed to be resident Mø since the organs they were obtained from were first perfused with Hanks’ balanced salt solution (HBSS) including heparin to remove as much blood as possible so as to avoid contamination of blood monocytes in the cultures. Indeed, the four propagated Mø clearly expressed the general resident Mø marker F4/80, which is usually expressed at relatively high levels in resident Mø with reduced levels in adult monocyte-derived Mø [5]. The propagated liver Mø were likely derived from Kupffer cells because no other resident Mø colonise the liver at such a large population. The spleen Mø were likely red pulp Mø because F4/80 is a marker for red pulp Mø but not for other resident Mø (i.e. marginal zone Mø, metallophilic Mø, tingible Mø) in the spleen [15, 21]. Alveolar Mø are shown to be F4/80^low/+^ and CD11b^−/low^ cells, while lung interstitial Mø are shown to be F4/80^+^ and CD11b^+^ cells [15, 22, 23]. We removed the alveolar Mø from the lung by bronchoalveolar lavage before collecting cells for Mø propagation, and the lung Mø were clearly F4/80- and CD11b-positive. A recent study by genetic lineage tracing revealed that lung interstitial Mø were derived from both foetal Mø in the yolk sac and adult monocytes differentiated in the bone marrow [24]. Very recently, it has also been shown that lung interstitial Mø derived from the bone marrow were slowly replaced by adult monocytes [25]. Thus, the propagated lung Mø were possibly lung interstitial Mø derived from foetal Mø. Although we used the brain tissue after removing the meninges to prevent contamination by meningeal Mø, we could not conclusively define the brain Mø as microglia based on the Mø marker expression patterns observed in flow cytometric analysis. The markers used in the present study [15, 26, 27] have been employed for identification of microglia, while brain Mø such as microglia and perivascular Mø commonly express these Mø markers. Most previous studies have regarded brain Mø appearing as small round cells in the top cell layer in primary co-culture as microglia based on expression of the general Mø markers [13, 28, 29] as shown in the present study. Recently niche signals and the signal-dependent transcription factors nourishing resident Mø of embryonic origin including Kupffer cells, red pulp Mø, alveolar Mø, and microglia have been revealed [30]. Thus further studies examining the expression of the transcription factors can elucidate detailed properties of the propagated liver, spleen, lung and brain Mø, and possibly determine the origin of these cells.

The successful propagation of four different Mø in vitro may further indirectly demonstrate the local proliferation property of resident Mø by self-renewal at steady state in vivo. Moreover, we found that all four Mø did not substantially propagate in bacteriological Petri dishes during the isolation process, where most of the Mø did not come into contact with cell aggregates consisting of stromal cells. This indicates that stromal cells of the respective organs might be indispensable for proliferation of the resident Mø, and that contact of the stromal cells with the resident Mø is possibly related to the production of cytokines such as CSF-1 and CSF-2, which promote the proliferation of resident Mø. This presumption is partly supported by the flow cytometry results. CSF-1 receptor (CD115) was clearly expressed in the liver, spleen, and lung Mø, but weakly/faintly expressed in the brain Mø, whereas CSF-2 receptor (CD116) expression was faint in the liver and brain. Further in vitro studies are required to examine the relation of contact of the stromal cells with the Mø to the production of those cytokines promoting the Mø proliferation. We speculated that co-cultures of Mø with organ-specific stromal cells likely mimic the niches for resident Mø in vivo, except for the co-culture of the brain cells since the propagation of brain Mø clearly decreased in the subculture after more than three passages. Stromal cells of the respective organs might exert an influence on the expression patterns of the Mø markers, as observed by flow cytometry, which were subtly different among the liver, spleen, lung, and brain Mø. These effects and the specific mechanism can be elucidated in further studies examining the effects on Mø expression properties by co-culture of resident Mø of respective organs with stromal cells of different origins. Moreover, co-culture with nerve cells is likely necessary for sustained propagation of the brain Mø in the subculture beyond three passages, because nerve cells are a source of IL-34, which is a ligand for the CSF-1 receptor [31].

## Conclusions

To the best of our knowledge, this is the first study to demonstrate the propagation of resident Mø colonising the spleen and lung. Moreover, resident Mø colonising the liver and brain have not previously been successfully propagated by subculture. Thus, this is the first study to clearly provide a practical Mø propagation method applicable to resident Mø of diverse tissues and organs. Recent studies have reported the potential use of Mø transplantation for the treatment of certain diseases [32–34]. For example, precursor origin cells such as yolk sac Mø and foetal monocytes, as well as mature resident Mø collected from certain organs efficiently colonised the corresponding organ after transplantation, while the mature resident Mø collected from other organs did not [34]. Thus, it is expected that resident Mø propagated by the present method can be applied to generate a sufficient quantity of cells as useful sources of transplantation to their corresponding organs in order to treat diseases.

## Methods

### Co-culture of macrophages with stromal cells obtained from the liver, spleen, lung, and brain

Stromal cells from the respective organs were harvested from specific-pathogen-free ICR male mice. The mice obtained from Japan SLC, Inc. (Hamamatsu, Japan) had been maintained under a standard housing condition in clean-grade environment on a 12-h light-dark cycle, and fed with standard diet and water ad libitum. In total, 18 mice were used. The animal experimentation protocol was approved by the Animal Research Committee of the Osaka Prefecture University. All experiments were performed in accordance with relevant guidelines and regulations of the Osaka Prefecture University.

Mice were sacrificed with an overdose of pentobarbital injected intraperitoneally (150 mg/kg body weight; Somnopentyl, Kyoritsu Seiyaku, Tokyo, Japan), and then intracardially perfused with Ca/Mg-free HBSS (Sigma-Aldrich, St Louis, MO, USA) supplemented with 50 U/mL heparin (Mochida Pharmaceutical, Tokyo, Japan) to remove the blood. The liver, lung, and spleen from 8-week-old mice and the brain from 4-week-old mice were aseptically dissected and immediately dipped in ice-cold HBSS. The gallbladder from the liver, adipose tissues around the splenic and pulmonary hilum from the spleen and lung, respectively, and the meninges, brainstem, and cerebellum from the brain were then removed. The lung was also cleared of alveolar cells, including alveolar Mø, by bronchoalveolar lavage. A 23-gauge intravenous catheter was inserted into the trachea, and 1.6 mL HBSS was injected and immediately withdrawn a few times. Half to about one-third of the whole liver as well as the whole spleen, lung, and cerebrum was minced with a razor blade into approximately 1-mm^3^ pieces and transferred to 15-mL conical tubes containing cell dispersion enzyme solution: 12 mL of 0.5 mg/mL Collagenase Type IV (Sigma-Aldrich) for liver tissues; 7.5 mL and 10 mL of 0.5 mg/mL Collagenase Type IA (Sigma-Aldrich) for spleen and lung tissues, respectively; and 10 mL of 1.0 mg/mL dispase (Thermo Fisher Scientific, Waltham, MA, USA) for brain tissues in 20 mM HEPES (Dojindo, Kumamoto, Japan)-buffered HBSS containing 1 mM CaCl_2_. The tissues were then digested at 37 °C for 40–60 min under gentle stirring at 120 rpm with one change of the digestion solution until tissue pieces were no longer visible. After washing with HBSS, cell/tissue suspensions were further dispersed by pipetting. The suspensions were sedimented at 100×*g* for 5 min (Model 2410, Kubota, Tokyo, Japan) and resuspended in HBSS to remove cell debris. Cells/tissues from half to one third of the whole liver, spleen, lung, and cerebrum per mouse were plated on three, one, three, and two 10 cm ø tissue culture dishes (AGC Techno Glass, Haibara, Japan), respectively. The dishes were coated for 2.5 h at 37 °C with or without collagen (Nitta Gelatin, Yao, Japan) in HBSS at 1.6 μg protein/cm^2^ for the liver, spleen, and lung cells and with poly-l-ornithine (Sigma-Aldrich) in HBSS at 1.9 μg protein/cm^2^ for the brain cells. Coated dishes were used for primary brain cell culture and in some cases for primary liver, spleen, and lung cell culture, but not for subculture. Cells were cultured in 12 mL DMEM (Sigma-Aldrich) containing 10% FBS (Sigma-Aldrich), 100 U/mL penicillin, and 100 μg/mL streptomycin (pen/strep; Sigma-Aldrich) (DMEM-FBS) for the liver, spleen, and lung as well as 12 mL DMEM/Ham’s nutrient mixture F-12 containing 10% FBS and pen/strep (DMEM/F12-FBS) for the brain. Cells were maintained in a humidified 5% CO_2_/95% air incubator at 37 °C. The medium was changed after a few hours and again after 1 day to remove non-adherent cells and cell debris, and thereafter every 4–6 days until dishes were covered by multi-layered cells composed of Mø and other stromal cells such as fibroblasts or astrocytes. Over-confluent cells were detached by 0.1% trypsin/1 mM EDTA in HBSS at 37 °C for 10–15 min followed by pipetting. Subsequently, cells at a dilution ratio of 1:3 for the liver, spleen, and lung and at 1:2 for the brain were subcultured or frozen at − 80 °C in a cell suspension with Bambanker (Nippon Genetics, Tokyo) as a cryopreservative and maintained in the same medium until they became over-confluent again.

### Separation of macrophages from stromal cells in co-culture

Co-cultured, over-confluent cells obtained from the liver and lung up to five passages, from the spleen up to eight passages, and from the brain up to two passages were used for separation of Mø. Cells harvested from a 10-cmø tissue culture dish at over-confluence were seeded in a 10-cmø bacteriological Petri dish (As One, Osaka, Japan) containing 10 mL DMEM-FBS. After one to a few days, when the Mø selectively adhered onto the dish surface and stromal cells formed aggregates floating in the medium, the cells were washed with conditioned media to remove cell aggregates. The adherent cells were then detached by 5 mL of 5 mM EDTA in 10 mM HEPES-buffered HBSS (HEPES-HBSS) at 37 °C for 10–15 min followed by pipetting. The cell suspension was passed through a cell strainer (BD Falcon, Franklin Lakes, NJ, USA) to remove cell aggregates, sedimented at 220×*g* for 5 min, suspended in phosphate-buffered saline (PBS) containing 1% bovine serum albumin (BSA; Sigma-Aldrich), 2 mM EDTA, and 0.01% NaN_3_ (BSA/EDTA-PBS), and the number of cells was calculated and used in experiments.

### Phagocytosis analysis with fluorescent beads

Cells (2.5 × 10^5^/0.5 mL DMEM-FBS) were placed in a 5-mL tube that was siliconised (Fuji-Rika Industries, Osaka, Japan) according to the manufacturer’s protocol to prevent adhesion to the tube wall. After addition of 1.0 μL fluorescent yellow-green-conjugated latex beads (mean diameter, 1.0 μm; Sigma-Aldrich), the cells were incubated at 37 °C for 2 h with gentle shaking at 18 rpm on a seesaw-type shaker (Wave SI slim; Taitec, Koshigaya, Japan), washed three times with HBSS, and plated on a 3.5-cmø glass-bottom dish (AGC Techno Glass) with 1.5 mL DMEM-FBS for ~ 2 h until almost all cells adhered to the surface. After fixation with 10% formalin (Kanto Chemical, Tokyo, Japan) in PBS for more than 10 min at room temperature (RT: 22–28 °C), phase-contrast and green fluorescence images of the same fields were captured using a 10× and 20× objective lens (IX71; Olympus, Tokyo, Japan). Cells engulfing more than two latex beads were denoted as Mø. We counted more than 700 cells per sample, and the percent of Mø in each organ was calculated from independent experiments (four mice and four experiments for the liver, spleen, and lung cells; six mice and six experiments for the brain cells). Statistical analyses were performed with the statistical software package Statcel (OMS Publishing Inc., Tokorozawa, Japan) implemented in Excel. Data are presented as means ± SD, and differences between groups were evaluated with unpaired *t*-tests. *P* values less than 0.05 were considered significant.

### Flow cytometry

Flow cytometry was used to determine the expression of Mø markers (CD11b, CD11c, CD68, CD86, CD115, CD116, CD169, CD184, CD206, F4/80, and MHC II) in cells segregated using bacteriological Petri dishes according to the method of Mukai et al. [35] with some modifications. Cells were prepared at a concentration of 1 × 10^6^ cells/mL in BSA/EDTA-PBS and fixed in 5% formalin in BSA/EDTA-PBS for 20 min at RT. After washing with BSA/EDTA-PBS, the cells were permeabilised in 0.2% saponin (Nacalai Tesque, Kyoto, Japan) in BSA/EDTA-PBS for 5 min at RT. To avoid non-specific Fc-gamma receptor-mediated binding of fluorochrome-conjugated antibodies, cell suspensions (~ 2.0 × 10^5^ cells/50 μL) were pre-treated with 0.5 μg of anti-mouse CD16/32 antibody (rat IgG2b; Tonbo Biosciences, San Diego, CA, USA) for 10 min at RT. To the 50 μL cell suspension, we added 0.5 μg FITC-conjugated anti-CD11b antibody (rat IgG2b; Tonbo), 0.25 μg APC-conjugated anti-CD11c antibody (hamster IgG; Tonbo), 0.15 μg FITC-conjugated anti-CD68 antibody (rat IgG2a; Miltenyi Biotec, Bergisch Gladbach, Germany), 0.125 μg FITC-conjugated anti-CD86 antibody (rat IgG2a; Tonbo), 0.5 μg FITC-conjugated anti-CD115 antibody (rat IgG2a; Tonbo), 0.1 μg APC-conjugated anti-CD116 antibody (rat IgG2a; R&D Systems, Minneapolis, MN, USA), 0.15 μg APC-conjugated anti-CD169 antibody (recombinant human IgG1; Miltenyi), 0.15 μg FITC-conjugated anti-CD184 antibody (recombinant human IgG1; Miltenyi), 0.25 μg APC-conjugated anti-CD206 antibody (rat IgG2b; Thermo Fisher Scientific, Waltham, MA, USA), 0.5 μg APC-conjugated anti-F4/80 antibody (rat IgG2a; Tonbo), and 0.25 μg APC-conjugated anti-MHC II antibody (rat IgG2b; Tonbo) according to the manufacturer’s instructions, followed by incubation for 10 min at RT. After washing, 20,000 or 30,000 cells were analysed for their expression characteristics using a flow cytometer (S3 Cell Sorter; Bio-Rad Laboratories, Hercules, CA, USA). As controls, we used cell suspensions that were pre-treated with the anti-mouse CD16/32 antibody and then treated with the same fluorochrome-labelled isotype control antibody of the same amount as the test antibody. Expression of marker molecules was determined from more than three independent experiments in cells propagated from each organ derived from more than three mice (four mice and four experiments for the liver, spleen, and brain Mø; three mice and three or four experiments for the lung Mø).

### M1/M2 polarisation by LPS plus IFN-γ and IL-4

Flow cytometry was also used to examine M1 and M2 polarisation in the spleen Mø as described above. The cells (8 × 10^5^) were plated on a 10-cmø bacteriological Petri dish with 10 mL DMEM-FBS. After 1 day of seeding, we added 20 ng/mL LPS (Sigma-Aldrich) plus 50 ng/mL IFN-γ (PeproTech, Rocky Hill, NJ, USA), 10 ng/mL IL-4 (Tonbo), or vehicle (HBSS) to the dish; 24 h later, the cells were detached by 5 mM EDTA. Cell suspensions (~ 2.0 × 10^5^ cells/50 μL) pre-treated with 0.5 μg of anti-mouse CD16/32 antibody were incubated with a mixture of 0.5 μg of FITC-conjugated anti-CD11b antibody and 0.25 μg of APC-conjugated anti-CD11c antibody, or 0.125 μg of FITC-conjugated anti-CD86 antibody and 0.25 μg of APC-conjugated anti-CD206 antibody.

## Data Availability

All data generated and analysed during this study are included in this published article.

## Abbreviations

CSF-1: Colony stimulating factor 1
CSF-2: Colony stimulating factor 2
DMEM: Dulbecco’s modified Eagle’s medium
F12: Ham’s nutrient mixture F-12
FBS: Foetal bovine serum
HBSS: Hanks’ balanced salt solution
IFN-γ: Interferon-γ
LPS: Lipopolysaccharide
MHC II: Major histocompatibility complex class II
Mø: Macrophages
PBS: Phosphate-buffered saline
pen/strep: penicillin and streptomycin
RT: Room temperature
